# Evaluation of Effects of Topical Estradiol Benzoate Application on Cutaneous Wound Healing in Ovariectomized Female Mice

**DOI:** 10.1371/journal.pone.0163560

**Published:** 2016-09-22

**Authors:** Kanae Mukai, Tamae Urai, Kimi Asano, Yukari Nakajima, Toshio Nakatani

**Affiliations:** 1 Faculty of Health Sciences, Institute of Medical, Pharmaceutical and Health Sciences, Kanazawa University, Kanazawa, Japan; 2 Department of Clinical Nursing, Graduate Course of Nursing Science, Division of Health Sciences, Graduate School of Medical Sciences, Kanazawa University, Kanazawa, Japan; University of Alabama at Birmingham, UNITED STATES

## Abstract

Estrogen promotes cutaneous wound healing in ovariectomized (OVX) female mice. However, the effects of topical estrogen application on wounds remain unclear. Therefore, the aim of this study was to compare the effects of topical estrogen application on wounds with standard treatment methods. Eight-week-old C57BL/6J female mice underwent OVX and received two full-thickness wounds four weeks later. Mice were divided into three groups: topical estradiol benzoate (EB) (0.75 μg/g/day) wound treatment, subcutaneous estradiol (E2) pellets (0.05 mg, 21 days), and topical E2 (0.01 g/day) skin application. Wound healing was observed until day 14. Wound area ratios were significantly smaller in the topical EB wound treatment group than in the subcutaneous E2 pellet group on days 1–14 (p < 0.05) and topical E2 skin application group on days 1–9 (p < 0.05). Neutrophil and macrophage numbers were significantly smaller in the topical EB wound treatment group than in the subcutaneous E2 pellet and topical E2 skin application groups on day 7 (p < 0.05). Moreover, the number of new blood vessels and ratio of myofibroblasts were significantly larger in the topical EB wound treatment group than in the subcutaneous E2 pellet and topical E2 application skin groups on day 7 (p < 0.05). These results demonstrate that the application of estrogen to wounds reduced inflammatory responses and promoted angiogenesis and wound contraction more than the two other standard treatment methods.

## Introduction

Cutaneous wound healing is a complex and tightly orchestrated response to injury, is carefully regulated at temporal and spatial levels [1], and involves three major stages: inflammation, proliferation, and remodeling. However, various factors play roles in cutaneous wound healing [2]. Recent studies reported that female sex hormones, in particular estrogen, affect cutaneous wound healing. Estrogen is the predominant steroid responsible for secondary sexual characteristics in girls and women and influences the function of all major organ systems within the body [3], and the ovary is the major source of estrogen production in the premenopausal period. Although estrogen is synthesized in steroidogenic cells of the ovary, estrogen is also synthesized in the skin. As the estrogen product decreases after the menopause, the skin is an important site of estrogen production [4]. However, in menopausal women, cutaneous wound healing was shown to be delayed and inflammatory responses prolonged following marked reductions in estrogen [5, 6]. These studies indicated that estrogen production in the skin is not sufficient to maintain cutaneous wound healing. Since delayed cutaneous wound healing increases health service costs [7], available treatments need to be performed in order to promote cutaneous wound healing.

One of these treatments, the application of estrogen is receiving increasing attention. Previous studies reported that the systemic administration of hormone replacement therapy (HRT) to menopausal women reversed delayed cutaneous wound healing [5], while the topical replacement of estrogen in healthy aged individuals reversed delayed cutaneous wound healing [6]. Therefore, in order to clarify the effects of estrogen on cutaneous wound healing, an ovariectomized (OVX) female rodent model is used and animal research has been performed. The administration of estrogen has been shown to promote cutaneous wound healing in 8-12-week-old OVX mice by decreasing neutrophil and macrophage numbers as well as the expression of TNF-α [8–13], by increasing Ym1-positive cell numbers [11] and the expression of TGF-β1 [5, 12], and by promoting collagen deposition [6, 9]. Moreover, we have evaluated the effects of estrogen on cutaneous wound healing upon delayed cutaneous wound healing using several OVX mice models. Our previous findings demonstrated that the administration of estrogen promoted cutaneous wound healing in 24- and 40-week-old OVX mice by reducing neutrophil and macrophage numbers and promoting re-epithelialization, collagen deposition, and wound contraction [14, 15]. We also recently reported that the administration of estrogen promoted the appearance of anti-inflammatory M2-like macrophages in protein malnutrition OVX mice [16].

In our previous studies, OVX mice were treated with the topical application of a 17β-estradiol (E2) gel (L’estrogel 0.06%; Bayer Yakuhin, Osaka, Japan) to the skin on the back, avoiding the wounds, every day after wounding [14–16]. In other studies, OVX mice were administrated a 0.05-mg, 21-d, slow-release E2 pellet (Innovative Research of America, Sarasota, FL) at the time of wounding by s.c. implantation [8–13, 17–21]. Many external agents have been applied to wounds and their effects on cutaneous wound healing have been evaluated [22–27]. However, the effects of topical estrogen application on wounds currently remain unclear. We hypothesized that the topical application of estrogen to wounds promotes cutaneous wound healing more than other standard treatment methods involving topical skin and subcutaneous applications. Therefore, the aim of the present study was to compare the effects of the topical application of estrogen on wounds with standard treatment methods in OVX mice.

## Materials and Methods

### Animals

Eighty-five C57BL/6 female mice aged 7 weeks (Sankyo Lab Service Co., Tokyo, Japan) were used in experiments. They were caged individually in an air-conditioned room at 25.0 ± 2.0°C with lights on from 08:45 to 20:45, and water and chow were given freely. All animal experiments conducted in this study were reviewed and approved by the Kanazawa University Animal Experiment Committee, and carried out in accordance with the Guidelines for the Care and Use of Laboratory Animals of Kanazawa University, Japan (AP-153483). Mice were acclimated for 7 days before the initiation of surgery. At 8 weeks, mice were anesthetized by inhalational anesthesia using 1.5% isoflurane (Wako, Tokyo, Japan) in 1.5 L O_2_/min over a plastic tube mask and the dorsum was shaved. They then underwent OVX according to the OECD guidelines [28] under inhalational anesthesia using 2.5% isoflurane. They were divided into three groups: a topical estradiol benzoate (EB) wound treatment group, subcutaneous estradiol (E2) pellet group, and topical E2 skin application group.

### Wounding

At 12 weeks (4 weeks after surgery), under anesthesia with shaving, two circular full-thickness skin wounds (4 mm in diameter) including the panniculus carnosus muscle were made on both sides of the dorsum of the mouse with a Kai sterile disposable biopsy punch (Kai Industries Co. Ltd., Gifu, Japan). The wounds were covered with a hydrocolloid dressing (Tegaderm; 3M Health Care, Tokyo, Japan) to maintain a moist environment, and the mouse was then wrapped with sticky bandages (Skinergate^™^; Nichiban, Tokyo, Japan), which were changed every day.

### Exogenous estrogen administration

In the topical EB wound treatment group, EB (Estra-1,3,5(10)-triene-3,17β-diol 3-benzoate) (OVAHORMON^®^INJECTION; ASKA Pharmaceutical Co. Ltd., Tokyo, Japan) was applied at 0.75 μg/g/day to the wounds every day after wounding. EB was diluted at 0.75 μg/g in sesame oil (Wako Pure Chemical Industries Ltd., Tokyo, Japan). In the subcutaneous E2 (Estra-1,3,5(10)-triene-3,17β-diol) pellet group, a 0.05-mg, 21-d, slow-release E2 pellet (Innovative Research of a America, Sarasota, FL) was administrated at the time of wounding by s.c. implantation according to previous studies [8–13, 17–21]. In the topical E2 skin application group, E2 gel (L’estrogel 0.06%; Bayer Yakuhin, Osaka, Japan) was applied at 0.01 g/day to the skin on the back, avoiding the wounds, every day after wounding according to our previous studies [14–16]. The dose administered was selected with successful estrogen replacement confirmed by an enzyme immunoassay on plasma samples, vaginal smears, or uterine weights.

### Macroscopic observations

The day when wounds were made was designated as day 0, and the process of wound healing was observed from then until day 14 after wounding. Wound edges were traced on polypropylene sheets and photographs were taken every day. The traces on the sheets were captured with a scanner onto a personal computer using Adobe Photoshop Elements 11.0 (Adobe System Inc., Tokyo, Japan), and the areas of wounds were calculated using the image analysis software ImageJ (National Institutes of Health, Bethesda, Maryland, USA). The wound area is shown as the ratio of the wound area every day to the initial wound area on day 0 when the wound was made, according to our previous studies [14–16].

### Uterus assay and enzyme immunoassay analysis of 17β-estradiol (E2)

Mice were euthanized by an overdose of pentobarbital sodium administered i.p. on days 3, 7 11, and 14. The uterus was harvested according to the OECD guidelines [28] and its wet weight was measured using a precision balance (d = 0.001 g) on days 3–14. In the additional three OVX mice, the uterus was harvested, and the effects of estrogen administration were examined in the in topical EB wound treatment, subcutaneous E2 pellet, and topical E2 skin application groups. Plasma was prepared from each mouse’s blood, isolated through cardiac puncture, and frozen until the time of assay. Enzyme immunoassay analysis (EIA) was performed to determine the concentration of E2 in the plasma on day 14, according to manufacture’s instruction (KGE014, R&D systems Inc., Tokyo, Japan).

### Histological procedure

Mice were euthanized by an overdose of pentobarbital sodium administered i.p. on days 3, 7, 11, and 14 after wounding. The wound and surrounding intact skin were harvested and each wound and surrounding intact skin sample was bisected at the wound center. One-half of each wound was stapled onto polypropylene sheets to prevent over-contraction of the sample and fixed in 4% paraformaldehyde for 17 hours. Samples were dehydrated in an alcohol series, cleaned in xylene, and embedded in paraffin to prepare 5-μm-thick serial paraffin sections. The remainder of each wound was embedded in tissue-Tek OCT (Sakura Finetek, Japan) before fixing to prepare 5-μm-thick serial ice sections. At least 3 serial paraffin sections and 2 serial ice sections near the center of the wound were obtained from one wound and stained according to the following methods. Five-μm-thick paraffin sections were subjected to hematoxylin and eosin (H&E) staining and immunohistologically stained with an anti-neutrophil antibody at a concentration of 1:100 (ab2557, Abcam Japan, Tokyo, Japan) to detect neutrophils and an anti-Mac-3 antibody at a concentration of 1:100 (550292, BD Pharmingen, Tokyo, Japan) to detect macrophages. Five-μm-thick ice sections were immunohistologically stained with an anti-α smooth muscle actin (α-SMA) antibody at a concentration of 1:300 (ab5694, Abcam Japan, Tokyo, Japan) to detect myofibroblasts and an anti-CD31 antibody at a concentration of 1:50 (550274, BD Pharmingen, Tokyo, Japan). Negative control slides were obtained by omitting each primary antibody. Immunohistological staining was performed as described below.

### Immunohistological staining

On paraffin sections, after deparaffinization and rehydration, antigen retrieval was achieved with Sodium Citrate Buffer (10 mM sodium citrate, 0.05% Tween20, pH 6.0) at approximately 100°C for 20 minutes. Endogenous peroxidase activity was blocked by 3% H_2_O_2_. Slides for an anti-mouse Mac-3 antibody were washed with phosphate-buffered saline (PBS), and slides for an anti-neutrophil antibody were washed with 0.3% Triton X-100 in PBS. Slides were then incubated with an anti-neutrophil antibody or Mac-3 antibody at a concentration of 1:100 in PBS at 4°C overnight. Slides were again washed. In order to detect primary antibodies, slides for the anti-mouse Mac-3 and anti-neutrophil antibodies were incubated with polyclonal rabbit anti-rat immunoglobulins/HRP (Dako North America, California, USA) at a concentration of 1:300 in 0.3% mouse serum (normal) (Dako North America, California, USA) in PBS at 4°C for 30 minutes. Slides were again washed, incubated in the Dako Liquid DAB+ Substrate Chromogen System (Dako North America, California, USA) for 5 minutes or until staining was detected at room temperature, and then counterstained with hematoxylin for 1 minute. All slides were rinsed in distilled water, dehydrated, cleared, and mounted for analyses.

On cryosections, 5-μm-thick sections were cut and fixed for 15 minutes in ice-cold acetone. In slides for the anti-α-SMA antibody, endogenous peroxidase activity was blocked by 0.3% H_2_O_2_ and slides were washed with 0.05% Tween-20 in PBS. Slides were then incubated with the anti-α-SMA antibody at a concentration of 1:500 in PBS at room temperature for 1 hour. Slides were again washed. In order to detect the primary antibody, slides for the anti-*α*-SMA antibody were incubated with the Dako Envision+ system HRP-labeled polymer anti-rabbit (ready to use) (Dako North America, California, USA) at room temperature for 30 minutes. Slides were again washed, incubated using the Dako Liquid DAB+ Substrate Chromogen System for 5 minutes or until staining was detected at room temperature, and then counterstained with hematoxylin for 1 minute. All slides were rinsed in distilled water, dehydrated, cleared, and mounted for analyses. Slides were incubated with the anti-CD31 antibody at a concentration of 1:50 in TBS at room temperature for 1 hour. Slides were washed with TBS. In order to detect the primary antibody, slides for the anti-CD31 antibody were incubated with goat polyclonal to Rat IgG-H&L (AP) (Abcam Japan, Tokyo, Japan) at room temperature for 2 hours. Slides were again washed, incubated using the BCIP/ NBT substrate system (Dako North America, California, USA) for 10 minutes or until staining was detected at room temperature, and counterstained with methyl green for 1 minute. All slides were rinsed in distilled water and mounted for analyses.

### Microscopic observations

Images were imported onto a computer using a digital microscopic camera (DP2-BSW Olympus, Japan). In order to analyze neutrophil and macrophage numbers in the wound area, each positive cell was counted using the image analysis software ImageJ with a x40 objective at five sites of the wound: two sites near the two wound edges and three sites around the center of the wound. The areas of these five sites were calculated on the monitor of DP2-BSW and the total numbers of neutrophils and macrophages at the five sites were divided by the whole area of these five sites. In order to analyze the numbers of new blood vessels in granulation tissue, each new blood vessel was counted using the image analysis software ImageJ with a x20 objective at five sites of granulation tissue: two sites near the two wound edges and three sites around the center of the granulation tissue. The total number of new blood vessels at the five sites was divided by the total area of these five sites. The number of blood vessels in normal skin was the same as that analyzed above. Measurements of brown-colored myofibroblasts (myofibroblast pixels/total wound pixels) were performed using Adobe Photoshop Elements 11.0, according to our previous studies [14–16].

### Statistical analysis

Data are expressed as the mean ± SD and analyzed using JMP^®^ 12.1.0 (SAS Institute Inc., Cary, NC, USA). Comparisons of means among multiple groups were performed with a one-way ANOVA followed by post hoc pairwise comparisons using the Tukey-Kramer multiple comparison test. P *<* 0.05 was considered significant.

## Results

### Uterine weights and 17β-estradiol (E2) value

**Fig 1:**
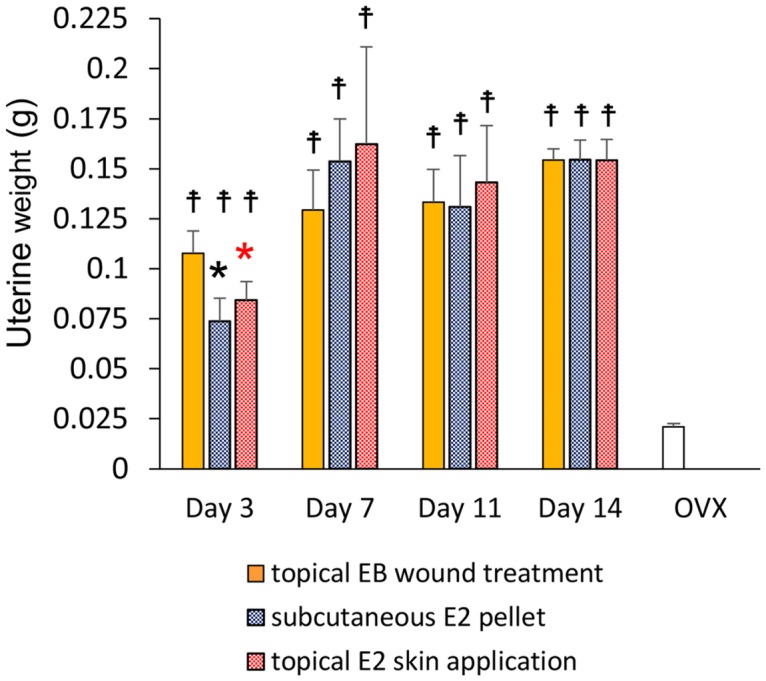
Uterine weight. Uterine weights are shown in box graphs. Values are expressed as the mean ± SD, n = 5–7 for each group, ANOVA, Tukey-Kramer *p<0.05 (in black): the topical EB wound treatment group versus the subcutaneous E2 pellet group, *p<0.05 (in red): the topical EB wound treatment group versus the topical E2 skin application group, ^☨^p<0.05: versus OVX.

We confirmed that the ovaries had been successfully removed in all groups. At each time point, uterine weight was significantly larger in the three groups than in OVX mice (p < 0.01). After the administration of estrogen, uterine weights in the three groups gradually increased until day 14 after wounding. Uterine weights increased more rapidly on day 3 in the topical EB wound treatment group than in the two other groups, and were also significantly larger in the topical EB wound treatment group than in the subcutaneous E2 pellet and topical E2 skin application groups on day 3 (p < 0.01) (Fig 1). On day 14, E2 value was 54.9±38.5 pg/mL in the topical EB wound treatment group, 38.1±20.6 pg/mL in the subcutaneous E2 pellet group, and 12.0±1.3 pg/mL in the topical E2 skin application group.

### Wound area

**Fig 2:**
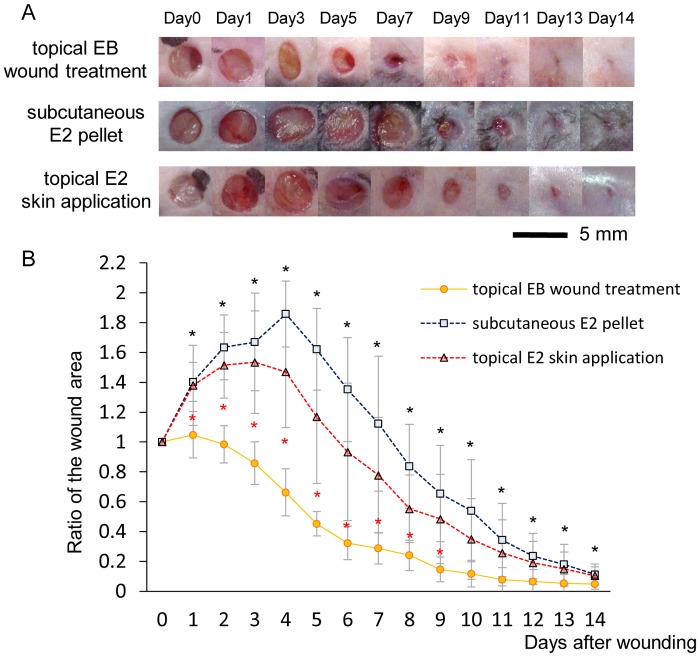
Macroscopic wound healing. (A) Wounds that were 4 mm in diameter were inflicted and healing was recorded by photography. Bar, 5 mm. (B) Ratios of wound areas to the initial area on day 0 are shown on line graphs for each day. Values are expressed as the mean ± SD, n = 10, ANOVA, Tukey-Kramer *p<0.05 (in black): the topical EB wound treatment group versus the subcutaneous E2 pellet group, *p<0.05 (in red): the topical EB wound treatment group versus the topical E2 skin application group.

In the subcutaneous E2 pellet and topical E2 skin application groups, wound areas increased for 5 days and then rapidly decreased until day 11, after which they decreased gradually until day 14 (ratio of the wound area to the initial wound area on day 14: 0.11 ± 0.07 in the subcutaneous E2 pellet group and 0.10 ± 0.06 in the topical E2 to the skin group). On the other hand, in the topical EB wound treatment group, wound areas only increased for 1 day and then rapidly decreased until day 9, after which they decreased gradually until day 14 (0.04 ± 0.03). The wound area ratio was significantly smaller in the topical EB wound treatment group than in the subcutaneous E2 pellet group on days 1–14 (p < 0.05) and topical E2 skin application group on days 1–9 (p < 0.05) (Fig 2A and 2B).

### Neutrophils and macrophages

**Fig 3:**
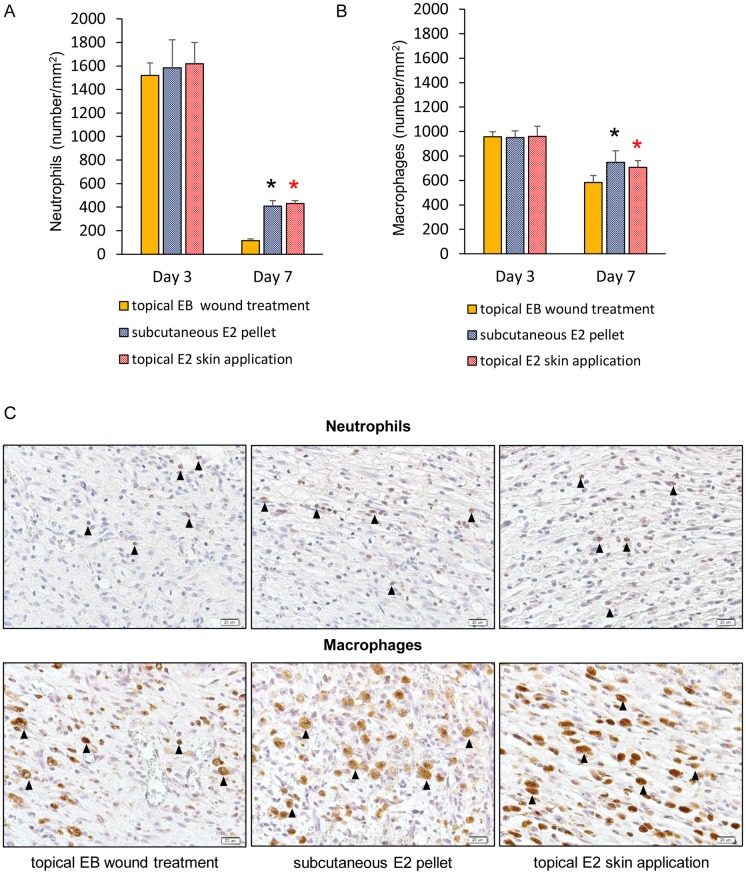
Neutrophils and macrophages. (A) The number of neutrophils per mm^2^ and (B) number of macrophages per mm^2^ are shown in box graphs. Values are expressed as the mean ± SD, n = 5–6 for each group, ANOVA, Tukey-Kramer *p<0.05 (in black): the topical EB wound treatment group versus the subcutaneous E2 pellet group, *p<0.05 (in red): the topical EB wound treatment group versus the topical E2 skin application group. (C) Neutrophils (arrows) stained with an anti-neutrophil antibody and macrophages (arrows) stained with an anti-Mac-3 antibody were observed in wound tissue on day 7. Bar, 20 μm.

A large number of neutrophils was observed in wounds in all groups on day 3 and then rapidly decreased until day 7, particularly in the topical EB wound treatment group. The number of neutrophils was significantly smaller in the topical EB wound treatment group than in the subcutaneous E2 pellet and topical E2 skin application groups (p < 0.05) (Fig 3A and 3C).

A large number of macrophages was also observed in wounds in all groups on day 3 and then decreased until day 7. The number of macrophages was also significantly smaller in the topical EB wound treatment group than in the subcutaneous E2 pellet and topical E2 skin application groups (p < 0.05) (Fig 3B and 3C).

### Angiogenesis and wound contraction

**Fig 4:**
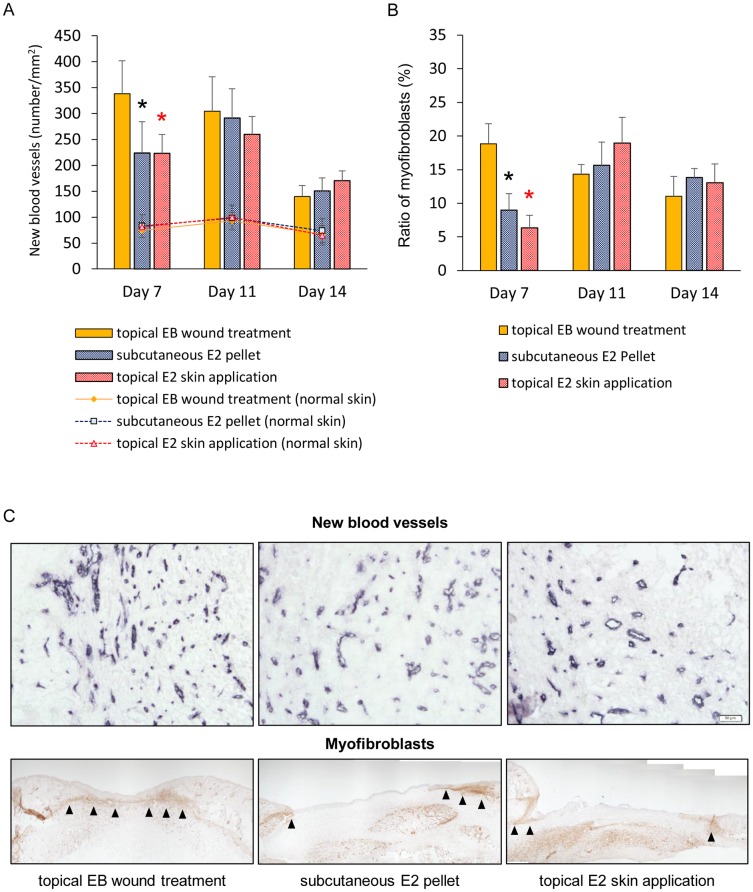
New blood vessels and wound contraction. (A) The number of new blood vessels per mm^2^ and (B) ratio of myofibroblasts (%) are shown in box graphs. Values are expressed as the mean ± SD, n = 5–6 for each group, ANOVA, Tukey-Kramer *p<0.05 (in black): the topical EB wound treatment group versus the subcutaneous E2 pellet group, *p<0.05 (in red): the topical EB wound treatment group versus the topical E2 skin application group. (C) New blood vessels stained with an anti-CD31 antibody (bars, 50 μm) and myofibroblasts (arrows) stained with an anti-α-SMA antibody (bars, 200 μm) were observed in granulation tissue on day 7.

In the topical EB wound treatment group, many new blood vessels were observed in granulation tissue on day 7 and gradually decreased in number from days 7 to 14. On the other hand, in the subcutaneous E2 pellet and topical E2 skin application groups, new blood vessels were observed in granulation tissue on day 7, they increased in number until day 11, and then decreased in number until day 14. The number of new blood vessels was significantly larger in the topical EB wound treatment group than in the subcutaneous E2 pellet and topical E2 skin application groups on day 7 (p < 0.05). Otherwise, the number of blood vessels in normal skin was the same in all groups on days 7 to 14 (Fig 4A and 4C).

In the topical EB wound treatment group, a large number of myofibroblasts was observed in granulation tissue on day 7 that formed bridge-like structures across the wound, and gradually decreased in number from days 7 to 14. On the other hand, in the subcutaneous E2 pellet and topical E2 skin application groups, myofibroblasts were observed in granulation tissue on day 7, increased in number until day 11, formed bridge-like structures across the wound, and then decreased in number until day 14. The ratio of myofibroblasts was significantly larger in the topical EB wound treatment group than in the subcutaneous E2 pellet and topical E2 skin application groups on day 7 (p < 0.05) (Fig 4B and 4C).

## Discussion

In the present study, we compared the effects of the topical application of estrogen on wounds with two other standard treatment methods. According to our hypothesis, cutaneous wound healing is promoted more by the effects of the topical application of estrogen to wounds than by the two other standard methods, namely, topical application to the skin on the back, avoiding the wounds [14–16], and the subcutaneous administration of a pellet at the time of wounding by s.c. implantation [8–13, 17–21]. Wound area ratios were significantly smaller in the topical EB wound treatment group than in the subcutaneous E2 pellet group on days 1–14 and topical E2 skin application group on days 1–9. This result shows that the topical application of estrogen to wounds reduced the wound area more than the two other standard methods during the whole process of healing. The effects of the topical application of estrogen to wounds may be related to early increases in uterine weights. Uterine weights were significantly larger in the topical EB wound treatment group than in the subcutaneous E2 pellet and topical E2 skin application groups on day 3. Successful estrogen replacement was confirmed by a gain in uterine weight [14–16]. Therefore, early successful estrogen replacement by the topical application of estrogen to wounds may be responsible for the effects observed.

The wound area did not increase in the inflammatory phase by the topical application of estrogen to wounds. The inflammatory phase is regarded as a critical period in cutaneous wound healing that is essential for clearing contaminating bacteria and creating an environment that is conducive to subsequent events involved in tissue repair and regeneration [29–31]. In the present study, the wound area only increased for 1 day in the topical EB wound treatment group, but increased for 5 days in the subcutaneous E2 pellet and topical E2 skin application groups. We previously demonstrated that cutaneous wound healing involves increases in the wound area in the inflammatory phase followed by gradual decreases [14–16, 26, 27]. Moreover, as an evaluation outcome of the inflammatory response, we also compared the numbers of neutrophils and macrophages. Neutrophils and macrophages are mainly active in the wound site in the inflammatory phase. Neutrophils prevent infection through phagocytic processes and propagate the inflammatory response by releasing cytokines [31, 32], and macrophages exhibit antimicrobial properties by releasing inflammatory cytokines [33]. The numbers of neutrophils and macrophages in wounds in the inflammatory phase have been shown to decrease in OVX mice administrated estrogen [8–13] and increases in the wound area in the inflammatory phase were slightly reduced in OVX female mice administrated estrogen [14] due to its anti-inflammatory effects [8–13]. In the present study, the numbers of neutrophils and macrophages were also significantly smaller in the topical EB wound treatment group than in the subcutaneous E2 pellet and topical E2 skin application groups on day 7. Therefore, these results suggest that anti-inflammatory effects were more prominent due to the topical application of estrogen to wounds than the two standard methods, resulting in a shorter inflammatory response.

Although the mechanism underlying the decrease in the number of inflammatory cells in cutaneous wound healing in the present study is still unknown, we hypothesize the cutaneous immune system to be responsible. The cutaneous immune system is integrated into the cutaneous neuro-endocrine system [34] through interaction with multiple pro-inflammatory and anti-inflammatory neuropeptides, cytokines, and hormones [35]. One component of the cutaneous neuro-endocrine system, the hypothalamic-pituitary-adrenal (HPA) axis, is fundamental as the body’s coordinator of responses to systemic and local stress [34, 36, 37]. At the local HPA axis, cytokines serve as communicators between the immunological and neuro-endocrine system, and a possible feedback loop in the HPA axis attenuates the initial pro-inflammatory responses, preventing excess inflammation [36]. A recent study reported that ultraviolet radiation (UV) exposure to the skin or skin cells enhanced cortisol production [38]. Furthermore, it was also reported that UV exposure up-regulated expression of anti-inflammatory cytokines [39], and these up-regulated anti-inflammatory cytokines also stimulated components of the HPA axis to enhance cortisol production [40]. These findings suggest that an external factor, such as UV exposure, can act as a trigger to activate the local HPA axis. Therefore, we speculate that topical application of estrogen to wounds may act as an external factor in the cutaneous neuro-endocrine system, similar to UV exposure, and prevent excessive inflammatory response. Moreover, we focused on the intracellular response. Estrogen signals act via two nuclear hormone receptors; estrogen receptor α (ER-α) and estrogen receptor β (ER-β) [36]. In mice skin, both receptors are widely expressed [17]. Previous study has reported that estrogen replacement in inflammatory cell specific ER-α null OVX mice (LysM-ERα) elevated local neutrophils and influx excessive macrophages [20], and macrophage recruitment in acute and chronic brain injury is mediated through ER-α [41]. It is also reported that estrogen replacement in LysM-ER-α OVX mice increased iNOS and reduced Arg1 [20] and ER-α KO mice display increased levels of pro-inflammatory chemokines during neuro-inflammation [42]. These studies suggest that inflammatory cell influx into the wound is mediated by estrogen signals via ER-α, and that cytokines are involved. Thus, we hypothesize that reduction of inflammatory cells after topical application of estrogen to wounds is mediated by estrogen signals via ER-α. Further research is needed to confirm these our theory. Furthermore, the reason why there were no significant differences between the topical application of estrogen to wounds and the two other standard treatment methods on day 3 remains unclear. Therefore, we also need to conduct further research in the near future.

Due to a shorter inflammatory response, wound contraction with the topical application of estrogen to wounds in OVX mice occurred faster than that by the two other standard treatment methods. In the present study, wound areas only increased for 1 day and then decreased rapidly until day 9 in the topical EB wound treatment group, whereas wound areas increased for 5 days and then decreased rapidly until day 11 in the subcutaneous E2 pellet and topical E2 skin application groups. This result is consistent with those obtained from immunohistological staining with the anti-α-SMA antibody. α-SMA is the most commonly used molecular marker for myofibroblasts [43] and α-SMA-positive myofibroblasts appear at the beginning of wound contraction and have intercellular adhesion molecules such as gap junctions linking them to each other [44–46]. In the present study, in the topical EB wound treatment group, many myofibroblasts were observed in granulation tissue on day 7, formed bridge-like structures across the wound, and then gradually decreased from days 7 to 14. On the other hand, in the subcutaneous E2 pellet and topical E2 skin application groups, myofibroblasts were observed in granulation tissue on day 7, increased in number until day 11, formed bridge-like structures across the wound, and then decreased in number until day 14. Moreover, the ratio of myofibroblasts was significantly larger in the topical EB wound treatment group than in the subcutaneous E2 pellet and topical E2 skin application groups on day 7. The events of wound contraction during cutaneous wound healing occurred as follows; initial phase, starting phase, increasing phase, decreasing phase, and scar phase [46]. In the starting phase, a few myofibroblasts appeared along the wound edge, and contraction started to occur, but weakly. At the increasing phase, a large number of myofibroblasts developed along the wound edge and wound bed, and formed bridge-like structures across the wound. In this phase, wound contraction is the strongest. In the decreasing phase, myofibroblast numbers in the wound bed markedly decreased, and, thus, the bridge-like structure disappeared. In this phase, wound contraction gradually weakens. Our results clearly show that the appearance of myofibroblasts in granulation tissue and formation of bridge-like structures occurred faster in the topical EB to wound treatment group than in the subcutaneous E2 pellet and topical E2 skin application groups. Therefore, wound contraction appears to be promoted more by the topical application of estrogen to wounds than the two other standard treatment methods. Moreover, with shortening of the inflammatory response, angiogenesis caused by the topical application of estrogen to wounds in OVX mice also occurred faster than the two other standard treatment methods. Estrogen may decrease vasoconstriction due to improvements in NOS signaling [47]. Functional impairments in the ERα/NOS-3 signaling network in OVX diabetic animals were partially restored by the administration of 17β-estradiol [48]. In an *in vitro* study, the capacity of endothelial cells for tube formation decreased in the presence of a high concentration of glucose (30 mM) and was rescued by a specific ERβ antagonist (PTHPT) [49]. These findings show that female sex hormones play important roles in angiogenesis. In the present study, the number of new blood vessels was significantly larger in the topical EB wound treatment group than in the subcutaneous E2 pellet and topical E2 skin application groups on day 7. Therefore, angiogenesis appears to have been promoted more by the topical application of estrogen to wounds than by the two other standard treatment methods. These results indicate that the topical application of estrogen to wounds promotes wound contraction and angiogenesis more than the two other standard treatment methods.

In summary, we found that the topical application of estrogen to wounds reduced the inflammatory response and promoted angiogenesis and wound contraction more than the two other standard treatment methods. Therefore, the topical application of estrogen to wounds is the most suitable treatment method of estrogen on cutaneous wound healing in OVX mice. We previously reported that the administration of estrogen promoted the appearance of anti-inflammatory M2-like macrophages in protein malnutrition OVX mice; however, it did not promote cutaneous wound healing with a low-protein diet [16]. These findings suggest that the administration of estrogen cannot promote cutaneous wound healing in malnutrition OVX mice. However, more than 50% of the elderly in hospitals and institutions were reported to be malnourished or at risk of malnutrition in developed countries [50, 51], and malnutrition has been shown to increase in Japan as the level of required care becomes high [51]. Therefore, we need to conduct further research in the near future in order to clarify whether the topical application of estrogen to wounds promotes cutaneous wound healing in malnutrition OVX mice.
